# Case report: bullous pemphigoid development underlies dystrophic epidermolysis bullosa disease worsening

**DOI:** 10.3389/fimmu.2022.929286

**Published:** 2022-07-29

**Authors:** Giovanni Di Zenzo, Giovanna Floriddia, Sabrina Rossi, Feliciana Mariotti, Alessia Primerano, Angelo Giuseppe Condorelli, Biagio Didona, Daniele Castiglia

**Affiliations:** ^1^ Laboratory of Molecular and Cell Biology, Istituto Dermopatico dell’Immacolata, IDI-IRCCS, Rome, Italy; ^2^ Pathology Unit, Department of Laboratories, Bambino Gesù Children’s Hospital, IRCCS, Rome, Italy; ^3^ Genodermatosis Unit, Genetics and Rare Diseases Research Division, Bambino Gesù Children’s Hospital, IRCCS, Rome, Italy; ^4^ Rare Skin Disease Center, Istituto Dermopatico dell’Immacolata, IDI-IRCCS, Rome, Italy

**Keywords:** pemphigoids, autoimmunity, genodermatosis, collagen VII, disease severity and modifiers

## Abstract

Autoimmune response to cutaneous basement membrane components superimposed on a genetic skin fragility disease, hereditary epidermolysis bullosa (EB), has been described, but its effects on disease course remain unclear. We report a 69-year-old individual with congenital skin fragility and acral trauma-induced blistering that had suddenly worsened with the onset of severe itch and diffuse spontaneous inflammatory blisters. Next-generation sequencing identified compound heterozygous null and missense *COL7A1* mutations, allowing the diagnosis of recessive dystrophic EB. However, the patient’s clinical history prompted us to investigate whether he might have developed a pathological autoimmune response against basement membrane components. Tissue-bound and circulating IgG antibodies to the major bullous pemphigoid (BP) antigen, BP180, were detected in the patient’s skin and serum, respectively, consistent with a diagnosis of BP. Corticosteroid therapy was initiated resulting in remission of BP manifestations. EB patients presenting rapid disease worsening should be investigated for the development of a concomitant autoimmune blistering disease.

## Introduction

Hereditary epidermolysis bullosa (EB) is a clinically and genetically heterogeneous group of disorders characterized by skin and mucous membrane fragility leading to trauma-induced blistering (1). Four major EB types are recognized based on the level of blister formation: EB simplex (EBS), typified by cleavage in basal keratinocytes; junctional EB (JEB) and dystrophic EB (DEB), characterized by separation within the lamina lucida and below the lamina densa of the cutaneous basement membrane zone (BMZ), respectively; and Kindler EB, where multiple cleavage levels are typically observed (2). Sixteen EB causative genes, mainly encoding structural proteins that ensure epithelial–mesenchymal adhesion, have been identified to date. Specifically, mutations in basal keratins 5 and 14, the hemidesmosomal plakins BP230 (dystonin) and plectin, underlie different EBS subtypes, while JEB is due to mutations in genes encoding the transmembrane hemidesmosomal proteins BP180 (collagen XVII) and integrin α6β4 as well as the epithelial laminin-332 chains. However, mutations in a single gene, *COL7A1*, encoding type VII collagen (C7), the major constituent of anchoring fibrils, are responsible for both dominant and recessive DEB, which is characterized by a wide spectrum of disease phenotypes ranging from localized forms, with acral skin lesions and nail dystrophies only, to severe variants with generalized mucocutaneous involvement, chronic ulcers, mitten deformities, and early onset of aggressive squamous cell carcinomas (1, 2).

Pemphigoid diseases are a group of subepithelial autoimmune blistering conditions affecting the skin or mucous membranes and typified by circulating and tissue-bound autoantibodies directed to BMZ components, the same proteins mutated in inherited EB (3). The commonest form is bullous pemphigoid (BP) caused by autoreactivity against BP180. Pathogenicity of anti-BP180 autoantibodies implies the formation of subepidermal blisters occurring within the lamina lucida of the cutaneous BMZ, the same cleavage plane of JEB (4).

Though having a distinct etiopathogenesis from pemphigoid diseases, recessive dystrophic EB (RDEB) presents immunological alterations, which may be related to anomalies of both innate and adaptive immune systems (5). In fact, deficiency of matrix proteins like laminin-332 and C7, in both thymus and lymph nodes, could affect T- and B-cell education (5). Moreover, C7 deficiency in secondary lymphoid organs makes skin and wounds of patients more susceptible to bacterial infections and dysbiosis, which could interfere with wound healing and trigger inflammatory cytokine production (5, 6). In this context, it has been reported that especially RDEB patients have high levels of proinflammatory cytokines (7). In addition, a large fraction of DEB patients (>70%) naturally develop circulating autoantibodies against BMZ proteins, as detected by ELISA and/or immunoblotting assays [(antibody titer between 9 and 96 U/ml for BP180 antigen and between 11 and 40 U/ml for BP230, using MBL ELISA kit, Nagoya, Japan; cutoff value 9 U/ml for both antigens; 40% is the proportion of patients with at least one positive BP180/230 antigen test (see reference 11)] (8–11). However, almost all of these patients lack the major immunodiagnostic criterion for an autoimmune bullous disease, i.e., a linear deposition of immunoglobulins and/or complement along the dermal-epidermal junction, as detected by direct IF. Moreover, their circulating autoantibodies do not bind to human skin, as detected by indirect immunofluorescence. Of note, autoreactivity to BMZ components has not been associated with changes in DEB clinical manifestations, and thus its role in disease course remains debated (8–11). Herein, we report an adult individual affected by localized RDEB who experienced a rapid worsening of his skin manifestations due to the development of BP.

## Case report

**Figure 1:**
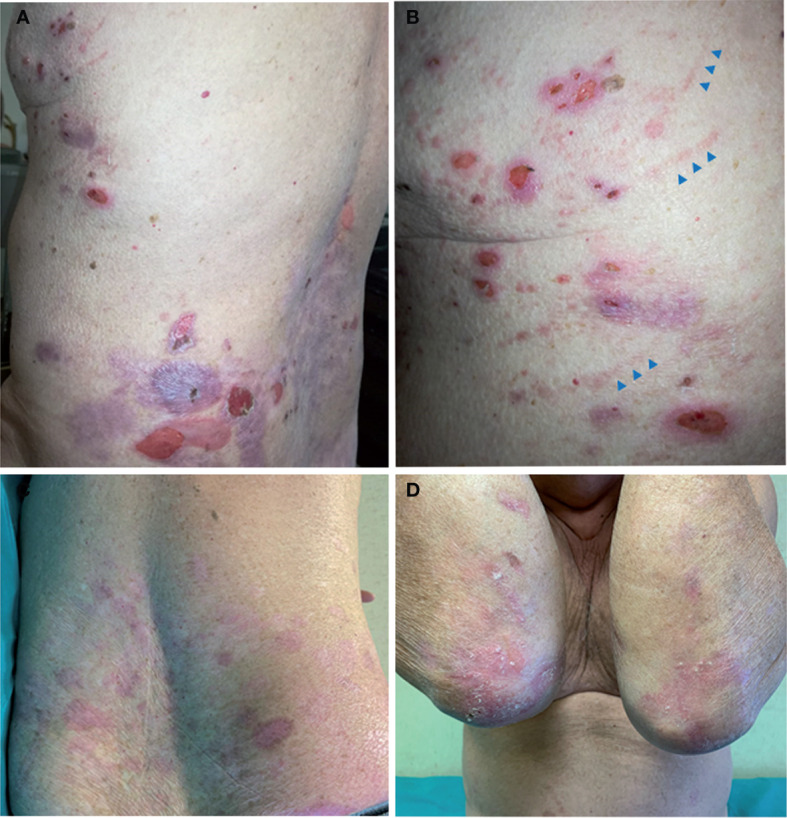
Clinical findings. **(A, B)** Patient clinical presentation at age 68 following disease worsening: blisters, erosions, and crusts with an erythematous halo are scattered on the back and thorax. Note linear erythematous lesions (arrowheads) on the lateral thorax in **(B)** due to scratching. **(C, D)** Clinical manifestations at age 69 when the patient was on bullous pemphigoid (BP) remission under minimal corticosteroid therapy. Note the absence of active lesions on the trunk, while skin erosions and atrophic scarring with milia, typical of localized recessive dystrophic epidermolysis bullosa, are visible on elbows.

**Figure 2:**
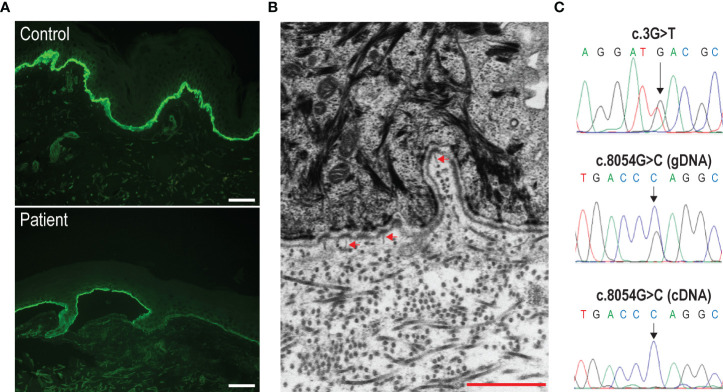
Immunofluorescence antigen mapping, and ultrastructural and molecular genetic findings. **(A)** Labeling intensity for type VII collagen is reduced in the patient’s skin (bottom panel) as compared to control skin (top panel). Bar = 40 µm. **(B)** Ultrastructural examination shows a few, hypoplastic anchoring fibrils (arrowheads) inserting onto the lamina densa of the cutaneous basement membrane zone in patient’s skin. Bar = 1 µm. **(C)** Detection of the c.3G>T (top panel) and c.8054G>C (middle panel) compound heterozygous mutations by Sanger sequencing of the patient’s genomic DNA (gDNA). The sequence of the complementary DNA (cDNA) across the c.8054G/C nucleotide position is also shown (bottom panel). Note that only transcripts carrying the c.8054C variation are detected.

**Figure 3:**
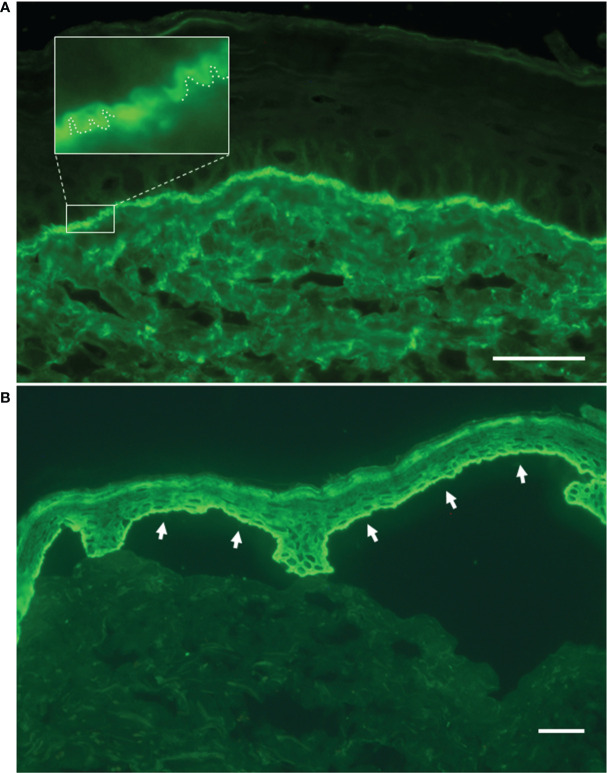
Direct and indirect immunofluorescence findings. **(A)** Linear deposit of IgG along the dermal–epidermal junction by direct immunofluorescence on patient’s perilesional skin. In the magnified inset, the ‘n’ serrated pattern further supports the bullous pemphigoid diagnosis. **(B)** Indirect immunofluorescence on salt-split skin with patient’s serum shows IgG binding to the roof of the split (white arrows). Bar = 40 µm.

Our proband was a 68-year-old man born to healthy non-consanguineous parents, with a life-long history of trauma-induced blisters mainly affecting the extremities, in the absence of mucosal involvement. The lesions healed with residual skin atrophy and milia formation. Mild toenail dystrophy was also present. EB was diagnosed in childhood. Skin fragility improved starting from adolescence with blistering almost exclusively localized to knees, elbows, pretibial areas, and hands and feet. The patient was referred to us at age 68 due to worsening of his skin fragility and blistering, accompanied by the onset of generalized severe itch in the last 1.5 years. He reported that disease changes manifested shortly after major emotional stress related to an episode of upper airway obstruction that happened to his wife. Physical examination revealed numerous blisters, erosions, and crusts surrounded by erythematous skin (**Figures 1A, B**). The lesions were diffusely distributed over the trunk, legs, and arms. In addition, he presented atrophic skin scarring with milia over elbows, knees, hand, and foot dorsa and toenail dystrophy. Perilesional skin biopsies for IF antigen mapping and electron microscopy were performed. IF mapping showed a slightly reduced expression of C7, which was localized to the roof in areas of dermal–epidermal detachment (**Figure 2A**). The expression of major BMZ protein components (integrin α6β4, BP180, and laminin-332) was comparable to that of control skin and also localized to the roof of the cleavage (not shown). Ultrastructural examination showed a reduced number of hypoplastic anchoring fibrils, which mostly lacked cross-banding (**Figure 2B**). To investigate the possibility of a superimposed autoimmune blistering disorder, direct IF (DIF) was also performed. It showed a continuous linear deposition of IgG along the BMZ (**Figure 3A**). Subsequent testing of the patient’s serum by indirect IF on salt split skin (IIF-SSS) revealed linear IgG along the roof of separated skin at a titer of 1:40 (**Figure 3B**). Moreover, commercial ELISAs based on BP antigens, BP180 and BP230 (MBL kit)), were positive (35.7 U/ml) and negative (0.9 U/ml), respectively. A commercially available ELISA for C7 (Euroimmun, Padua, Italy; cutoff value 20 UR/ml) proved negative (0.6 UR/ml). In parallel, the molecular diagnosis was obtained by massive parallel sequencing of the patient’s genomic DNA with an exome panel approach (TruSight One expanded, Illumina, San Diego, CA, USA). Datasets retrieved from the 16 EB genes revealed two variants in *COL7A1*: c.3G>T and c.8054G>C (**Figure 2C**, top and middle panels). The former is a known pathogenic mutation resulting in the start codon loss p.Met1?; the latter causes the missense change p.Arg2685Pro affecting the Y position of a Gly-X-Y triplet of the collagenous domain (12). Mutations were screened in the two healthy patient’s sons, each of whom was found to carry one of the two variants. The p.Arg2685Pro has not been previously published in the literature and is a known variant (rs886058630) found at the heterozygous state with an allele frequency ranging from 0.002% and 0.003% (TOPMed and gnomAD source). It was considered likely pathogenic according to American College of Medical Genetics and Genomics (ACMG) guidelines as i) it is located in a known functional protein motif/domain (PM1), ii) it is found at an extremely low frequency (PM2), iii) it is detected in *trans* with a known (p.Met1)? pathogenic variant (PM3), and iv) computational evidence (SIFT, PolyPhen, Mutation Taster, FATHMM, MetaLR, and REVEL) supports a deleterious effect on the gene product (PP3) (13). Reverse transcriptase (RT)-PCR analysis of the mRNA extracted from patient keratinocytes identified molecules transcribed only from the c.8054C allele (**Figure 2C**, bottom panel). This finding demonstrated that the mutation c.3G>T (p.Met1)? on the other allele strongly affects the mRNA biogenesis and/or stability, while the c.8054G>C change does not.

Based on the clinical and laboratory findings, a diagnosis of RDEB with concomitant BP was made. Therefore, prednisone treatment for BP at 0.6 mg/kg/day was started, resulting in BP disease control within 1 week, followed by gradual tapering. The patient is currently in remission for BP with minimal steroid therapy (10 mg/day) (**Figures 1C, D**).

**Figure 4:**
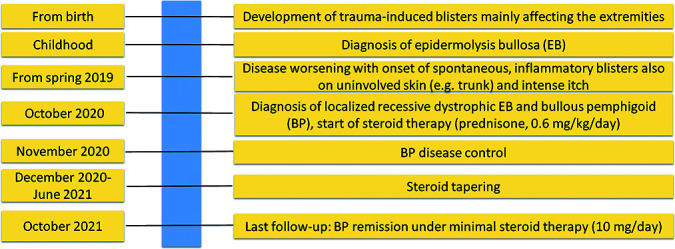
Timeline of patient’s clinical history, and diagnostic and therapeutic steps.

The timeline of the patient’s clinical history, diagnostic steps, and BP treatment is summarized in **Figure 4**.

## Discussion

Here, we report a patient with RDEB presenting a peculiar disease course owing to the onset of BP. Compound heterozygosity for p.Met1? and p.Arg2685Pro mutations in *COL7A1* are in line with the immunomapping and ultrastructural findings and conform to the genotype–phenotype correlation rule for localized RDEB (2). The p.Met1? is a null variant that has been previously reported in patients with absent C7 and severe RDEB in combination with frameshift mutations (12). The p.Arg2685Pro allows for stable mRNA transcripts and protein synthesis, as documented by RT-PCR and immunofluorescence analysis. This latter variant has not been previously published and was classified as likely pathogenic according to ACMG (13). Functionally, the p.Arg2685Pro substitution affects a Gly-X-Y repeat of the collagenic domain; thus, it is predicted to perturb C7 triple helix assembly and/or stability.

Autoimmune bullous diseases have been previously reported in extremely rare cases of hereditary EB, including two cases of DEB who both developed EB acquisita (EBA), which is typified by circulating and tissue-bound autoantibodies against C7 (14, 15). Our patient is the first example of BP occurring in RDEB. These three cases share a similar degree of DEB severity, which, before autoimmunity onset, was at the milder extremity of the disease phenotypic spectrum. Indeed, *COL7A1* mutations found in each of them predicted a mild and steady disease, in contrast to the worsening of the clinical manifestations, which they experienced in adulthood. The disease exacerbation was even more evident in the current localized RDEB case than in the two previously published patients with localized DEB (14, 15), as the blisters arose in previously unaffected body areas, such as the trunk, and were accompanied by severe pruritus. Disease extension and change were easily understood by the patient himself, representing the reason for dermatological consultation in our hospital. In addition, a recent study aimed at screening the occurrence of autoantibodies in a cohort of EB patients reported that two out of 35 cases (5.7%) showed laboratory findings consistent with a diagnosis of EBA, i.e., linear binding of IgG along the BMZ in DIF, dermal binding of IgG in IIF-SSS, and positive anti-C7 autoantibodies by ELISA (11). Differently from previously mentioned mild DEB cases, both patients were affected by severe RDEB with negative C7 immunostaining, and no change in skin manifestations was noticed. However, it cannot be excluded that the severity of the RDEB manifestations has masked a phenotypic worsening. Additionally, considering the extremely low amount, if any, of residual C7 in RDEB severe, it is possible that the autoantibody response may not result in an overt increase of skin fragility in these patients. Finally, disease worsening due to BP development occurred in another previously published adult patient affected by intermediate JEB due to mutations in the laminin β3 chain (16). Notably, in that case, DIF performed both on the original skin biopsy used for initial JEB diagnosis and on the skin biopsy obtained after the disease worsened proved negative and positive, respectively, documenting the causal relationship between the autoantibodies binding to the patient’s skin and the phenotype modification (16). Of note, in the patient here described corticosteroid therapy administered for BP rapidly resulted in remission of newly developed skin manifestations and concomitant severe itch, further supporting the role of the autoimmune response in disease worsening.

Our patient linked the disease worsening to a recent strong negative emotional stress. Precipitating factors for BP development have been identified in approximately 15% of patients (17). Emotional stress is often reported by patients as a trigger factor for various diseases including inflammatory and autoimmune skin conditions, such as vitiligo and pemphigus (18, 19). As neural, endocrine, and immune systems interact, emotional stress may be hypothesized as a trigger factor for BP onset in our RDEB patient (20). Moreover, most of the recognizable trigger factors for BP are related to local disruption of the cutaneous BMZ, indicating that structural alterations can modify the antigenicity of BMZ components with exposure to neoepitopes (17). Consistently, chronic injury and inflammation typical of RDEB, an inherent alteration of the immune system consequent to C7 loss, and the interplay between predisposing and provoking factors, such as aging, genetic predisposition, and emotional stress, could have played a role in the onset of BP in our patient (5, 6, 17). In conclusion, we suggest that the development of a pathological autoimmune response may occur in a subset of adult RDEB individuals as a result of a variable combination of genetic predisposition, chronic skin damage, underlying immunity defects, and specific triggers. In EB patients presenting unexpected disease worsening during adulthood, a DIF reaction on skin biopsy should be always performed to assess the possible development of a concomitant autoimmune bullous disease, which, if confirmed, prompts immunosuppressive and immunomodulatory treatments aimed at controlling the autoimmune response.

## Data availability statement

The datasets supporting the conclusions of this study are available from the corresponding author upon reasonable request. The raw datasets generated during the current study are not publicly available because it is possible that individual privacy could be compromised.

## Ethics statement

The studies involving human participants were reviewed and approved by IDI-IRCCS Ethics Committee (ID #222/1, 2007, and #578/1, 2019). The patients/participants provided their written informed consent to participate in this study. Written informed consent was obtained from the individual(s) for the publication of any potentially identifiable images or data included in this article.

## Author contributions

DC contributed to the writing and editing of the manuscript. DC, GD, and BD contributed to the conception. GF, FM, AP, AC, and SR contributed to the data collection. DC and GD conducted the supervision. All authors contributed to the article and approved the submitted version.

## Funding

This article was supported by the ‘Progetto Ricerca Corrente - 2020’ of the Italian Ministry of Health, Rome, Italy.

## Acknowledgments

We thank Naomi De Luca and Riccardo Mariani for their skillful technical support. IDI-IRCCS and Bambino Gesù Children’s Hospital, IRCCS, are healthcare providers of the European Reference Network for Rare and Undiagnosed Skin Diseases (ERN-Skin).

## Conflict of interest

The authors declare that the research was conducted in the absence of any commercial or financial relationships that could be construed as a potential conflict of interest.

## Publisher’s note

All claims expressed in this article are solely those of the authors and do not necessarily represent those of their affiliated organizations, or those of the publisher, the editors and the reviewers. Any product that may be evaluated in this article, or claim that may be made by its manufacturer, is not guaranteed or endorsed by the publisher.
